# Group living in highland tuco-tucos (*Ctenomys opimus*) persists despite a catastrophic decline in population density

**DOI:** 10.1371/journal.pone.0304763

**Published:** 2024-06-07

**Authors:** Eileen A. Lacey, Shannon L. O’Brien, Pablo A. Cuello, Mauro N. Tammone

**Affiliations:** 1 Museum of Vertebrate Zoology, University of California, Berkeley, Berkeley, California, United States of America; 2 Department of Integrative Biology, University of California, Berkeley, Berkeley, California, United States of America; 3 Animal Welfare Science Program, Lincoln Park Zoo, Chicago, Illinois, United States of America; 4 Instituto Argentino de Investigaciones de Zonas Áridas, CONICET-Universidad Nacional de Cuyo, Mendoza, Argentina; 5 Instituto de Investigaciones en Biodiversidad y Medioambiente (CONICET-UNComahue), Bariloche, Río Negro, Argentina; University of Pretoria, SOUTH AFRICA

## Abstract

Identifying the factors that favor group living is central to studies of animal social behavior. One demographic parameter that is expected to substantially shape spatial and social relationships is population density. Specifically, high population densities may favor group living by constraining opportunities to live alone. In contrast, low densities may allow individuals to spread out within the habitat, leading to a reduction in the prevalence or size of social groups. Abrupt changes in density following natural catastrophic events provide important opportunities to evaluate the effects of population density on patterns of spatial and social organization. As part of long-term studies of the behavioral ecology of a population of highland tuco-tucos (*Ctenomys opimus*) at Monumento Natural Laguna de los Pozuelos, Jujuy Province, Argentina, we monitored the demographic and behavioral consequences of a flood that inundated our study site during December 2012. Unlike most species of *Ctenomys* studied to date, highland tuco-tucos are group living, meaning that multiple adults share burrow systems and nest sites. Despite a post-flood reduction in population density of ~75%, animals present on the study site during the 2013 breeding season continued to live in multi-adult social units (groups). No differences between pre- and post-flood home range sizes were detected and although between-unit spatial overlap was reduced in 2013, overlap within social units did not differ from that in pre-flood years. Animals assigned to the same social unit in 2013 had not lived together during 2012, indicating that post-flood groups were not simply the remnants of those present prior to the flood. Collectively, these findings indicate that group living in highland tuco-tucos is not driven by the density of conspecifics in the habitat. In addition to enhancing understanding of the adaptive bases for group living in *Ctenomys*, our analyses underscore the power of catastrophic events to generate insights into fundamental aspects of social behavior.

## Introduction

Understanding the reasons for group living is a fundamental goal of behavioral biology [1, 2]. Both ecological and demographic factors are thought to play critical roles in determining whether animals live alone or in groups [3, 4]. For example, groups may form because individuals congregate around critical resources (e.g., food or shelter [5]) or because the presence of multiple conspecifics offers solutions to ecological challenges that cannot be achieved by lone animals (e.g., cooperative defense against predators [6]). At the same time, ecological or demographic conditions (e.g., shortage of suitable habitat or potential mates [7, 8]) may constrain dispersal, leading to the formation of groups as individuals accumulate in their natal areas [9, 10]. Because the precise combinations of factors favoring group living are likely complex and because ecological and demographic conditions are often intertwined [3, 11, 12], efforts to evaluate the effects of specific parameters on social organization can be challenging, particularly when studying free-living animals in which opportunities for controlled manipulations of key variables are limited [13]. Under these circumstances, catastrophic events (e.g., unexpected significant changes in environmental conditions) may serve as critical natural experiments that can be used to assess the roles of different ecological and demographic factors in promoting group living [14, 15].

One demographic factor that is expected to influence the tendency to live in groups is population density [16, 17]. When density is high, individuals may be forced to live together due to a shortage of unoccupied areas that allow survival and reproduction [3, 18, 19]. In contrast, when density is low, individuals may be able to distribute themselves more widely across the landscape, resulting in fewer groups and a greater number of lone animals [20–22]. Accordingly, how members of a population respond to changes in density may generate important insights into the adaptive bases for group living. While long-term studies can be used to examine relationships between behavior and typical changes in density [21, 23], abrupt catastrophic changes in population density offer important opportunities to evaluate the effects of more extreme fluctuations in this demographic parameter. Abrupt declines in density have been reported for multiple species following a variety of natural catastrophic events [24–28]. Although often unpredictable, such events provide potentially compelling information, particularly if the resulting densities fall outside the range of values typically experienced by members of a population.

The highland tuco-tuco (*Ctenomys opimus*) is a social species of rodent that occurs in the Puna ecoregion of the Andes Mountains [29, 30]. Like other members of the genus *Ctenomys*, highland tuco-tucos are subterranean, meaning that the animals live in extensive networks of underground burrows [31, 32]. Compared to most of its congeners, however, *C*. *opimus* spends a large proportion of time above ground, with animals regularly leaving their burrows to forage on surface growing vegetation. Although most aspects of the biology of this species have yet to be characterized, the behavior of *C*. *opimus* has been studied in the vicinity of Laguna de los Pozuelos, Jujuy Province, Argentina. While most of the > 60 described species of tuco-tucos are thought to be solitary [33, 34], radio telemetry analyses indicate that the population of *C*. *opimus* at Pozuelos is group living, meaning that multiple adults share a burrow system and subterranean nest site [35, 36]. Spatial and social relationships are dynamic, with group size and composition varying markedly between years [36]. At the individual level, group size tends to increase over time [36], indicating that age may contribute to an animal’s social relationships. Although kinship has not been quantified for members of the Pozuelos population, the overall variability in social behavior reported [36] suggests that kin relationships are unlikely to be a key determinant of group composition. Instead, O’Brien et al. [36] propose that fluctuations in local ecological and demographic conditions (e.g., food resources, population density) are the primary drivers of variability in the social behavior of this species. The effects of such parameters have not been examined quantitatively and thus studies of these animals provide an important opportunity to explore the ecological and demographic factors associated with the formation of social groups.

As part of long-term analyses aimed at identifying the adaptive bases for group living, we monitored spatial and social relationships among members of the population of *C*. *opimus* at Pozuelos from 2010 to 2014 (see also [36]). In December 2012, the area was flooded by the Río Cincel. This event killed most of the tuco-tucos on our study site, leading to a dramatic reduction in the density of this population. Accordingly, the flood offered an important opportunity to assess the effects of population density as well as several related demographic parameters (e.g., age- and sex-related mortality) on the social organization of these animals. If group living in *C*. *opimus* occurs due to constraints imposed by the presence of numerous conspecifics, then the apparent post-flood decline in density should have been associated with a reduction in the tendency for the animals to live together. In particular, lower population density may have allowed individuals to spread out within the habitat, leading to reduced overlap of home ranges and, potentially, a reduction in the prevalence and size of social groups. To test these predictions, we compared pre- and post-flood spatial relationships within the study population. We also compared pre- and post-flood values for several metrics of sociality, including group size and the proportion of animals living in groups. These analyses generate critical insights into the role of population density in shaping the social organization of highland tuco-tucos, with implications for understanding the adaptive bases for group living in other burrow dwelling rodents.

## Materials and methods

### Study site and study population

**Fig 1:**
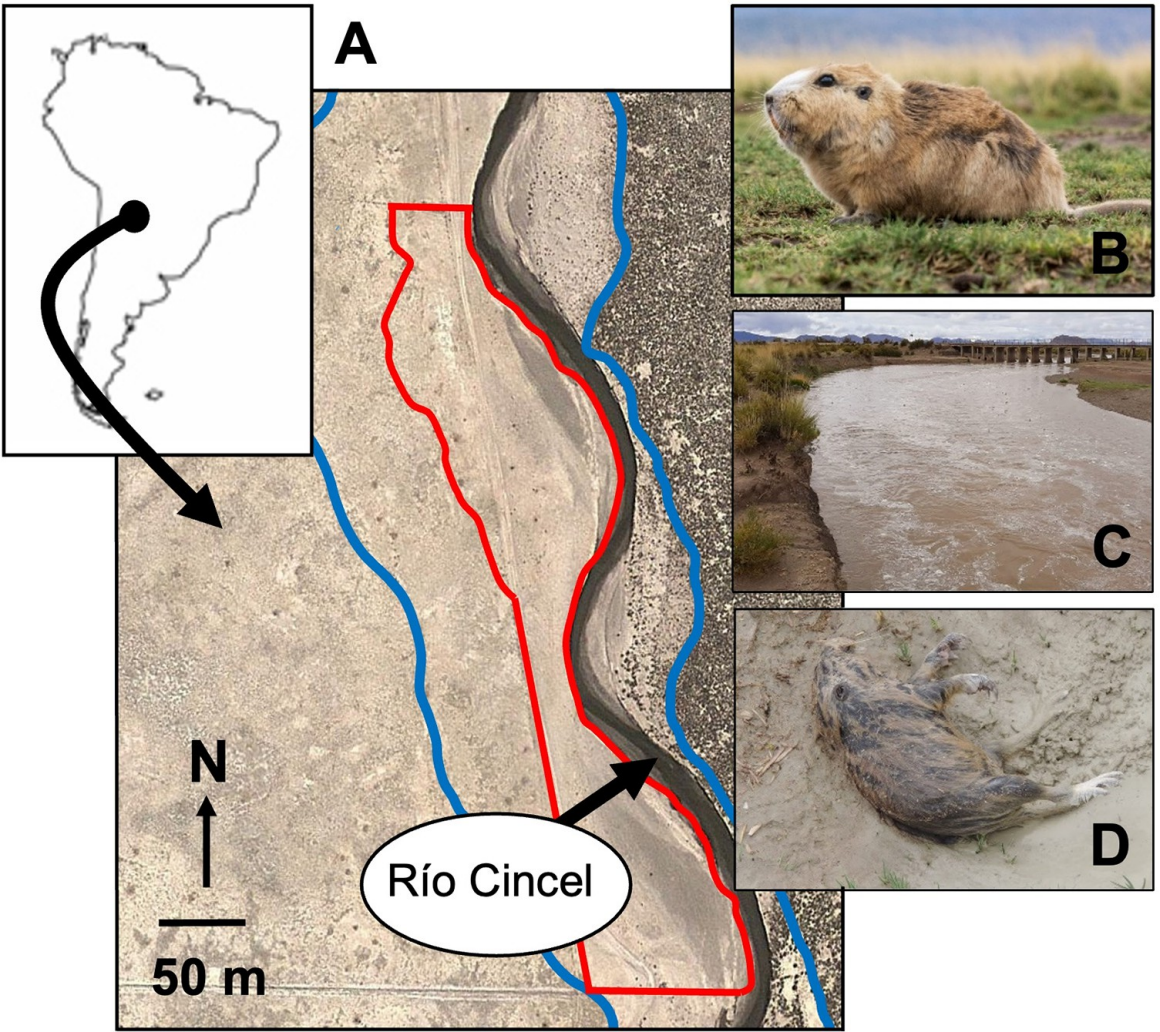
Study site at Monumento Natural Laguna de los Pozuelos, Jujuy Province, Argentina. In (A), the area outlined in red denotes the boundaries of the study site, which was located along the western bank of the Río Cincel. The area outlined in blue depicts the approximate extent of flooding by the river during December 2012. (B) An adult male highland tuco-tuco (*Ctenomys opimus*). (C) Photo of the Río Cincel looking south from the study site. (D) Dead highland tuco-tuco found on the study site after the flood waters receded. Photo credits: (A) Public Domain Map (https://openstreetmap.us/our-work/public-domain-map/), (B) Christian Irian, (C) and (D) Walter Arias, Cristian Maimani, and Santiago Nicolás Gorini.

The population of highland tuco-tucos (*Ctenomys opimus)* studied was located in Monumento Natural Laguna de los Pozuelos in Jujuy Province, Argentina (-22.469347, -65.994279, WGS 84). Pozuelos sits in a high Andean valley that is dominated by tola (*Parastrephia* sp.) shrubland punctuated by more open patches of salt grass (*Distichlis* sp.). Although tuco-tucos are typically abundant in salt grass, the animals rarely penetrate more than a few meters into the surrounding tola. The study site consisted of a ~ 3 ha area of salt grass habitat along the western bank of the Río Cincel (Fig 1). The site was bounded to the west by tola and to the north and south by the remnants of adobe walls used to enclose livestock. Accordingly, while the study site encompassed the east-west extent of the population, animals were distributed beyond the northern and southern limits of the site.

Annual precipitation at Pozuelos is typically ≤ 200 mm, with rainfall occurring primarily during the austral summer, from December to March [37]. Storms may be locally intense and can cause rapid changes in the level of the Río Cincel. In December 2012, multiple days of heavy rainfall caused the river to overflow its banks, covering the entire study site with water (Fig 1). The site remained flooded for approximately one week, after which the river receded to its typical boundaries, leaving the bodies of numerous dead tuco-tucos visible on the soil surface (Fig 1). This was the only such flood to affect the site during the years of this study and this event provided an unexpected but important opportunity to assess relationships between population density and the social organization of the study animals.

### Ethics statement

All procedures involving live animals were approved by the Animal Care and Use Committee at the University of California, Berkeley (AUP #2015-01-7054-3) and were consistent with the guidelines of the American Society of Mammalogists for the use of wild mammals in research [38]. Permission to conduct research at Pozuelos was provided under permit #IF-2019-98967871 issued by the Delegación Técnica NOA of the Administración de Parques Nacionales Argentinas. Members of the study population were generally captured only once per year. Trapping and handling procedures were designed to minimize the time that animals were restrained; typically, handling was completed and individuals were returned to their burrow systems within 15 minutes of capture. Subsequent collection of behavioral and spatial data was completed via visual observations of members of the study population.

### Capture and marking of study animals

Tuco-tucos on the study site were monitored annually from 2010 to 2014. Although the reproductive cycle of *C*. *opimus* has not been quantified in detail, our observations of the study population combined with information from park guards and local residents suggest that females produce litters during the late austral spring and early austral summer, with a potential second, less pronounced round of reproduction in the austral autumn. Field work was conducted during November-December of each year, which corresponds to the primary period of offspring production. The mean duration of each field season was 21.8 ± 4.2 days (n = 5 seasons). During each season, we attempted to capture all animals on the study site using tomahawk-style wire mesh traps baited with carrots. Traps were set during daylight hours and were monitored continuously while open; individuals were retrieved as soon as they were caught. Sex and body mass were recorded for each individual captured. For females, reproductive status (receptive, pregnant, lactating) was assessed based on visual inspection of the genitalia and mammae as well as palpation of the abdomen; because the testes of male tuco-tucos are never visible externally [39], the reproductive status of members of this sex could not be determined based on visual inspection of these animals. The criteria used to determine the relative age (subadult, adult) of each animal are described below.

Individuals captured for the first time were permanently marked by injecting a PIT tag (IMI-1000 tags, Biomedic Data Systems, Seaforth, DE) beneath the skin at the nape of the neck. PIT tags were read using handheld scanners (DAS 4007, Biomedic Data Systems, Seaforth, DE) and provided a reliable means of identifying animals across successive field seasons. To allow visual identification of individuals, human hair dye was used to mark the fur of each animal with a unique combination of colored patches; markings lasted ~ 3 weeks, meaning that individuals could be identified for the approximate duration of each field season. Because members of the study population emerged from their burrows numerous times per day and would often spend extended periods of time above-ground while foraging, fur markings provided an easy method of distinguishing individuals from one another. Additionally, fur markings allowed for ready detection of any animals that were not captured (i.e., lacked dye markings) during a given field season, thereby enabling more accurate estimates of annual population density. Finally, observations of visually distinct animals were used to characterize the home ranges of members of the study population (see below).

### Determining relative age

To characterize the age structure of the population and to evaluate potential age-specific effects of the flood on density or other demographic parameters, the relative age (subadult, adult) of each animal captured was determined based on the combination of body mass and either reproductive status (females) or pelage attributes (males). Females displaying evidence of reproductive activity (see above) were assumed to be adults. To determine if non-reproductive females tended to be significantly smaller (i.e., subadults), body masses for these animals were compared to those of reproductive females. Because body mass can vary due to multiple factors (e.g., age, sex, resource availability), we used a linear model to evaluate the relationship between mass and putative age. Body masses were not normally distributed and thus this model was based on a Poisson distribution, as implemented in the lme4 package in R [40]. Reproductive status was included as a fixed effect, with year included as a random effect. All females captured in two successive field seasons were reproductively active during their second year; because these animals were clearly adults during their second season, we also ran this model using data from second-year versus non-reproductive females captured during the same field season.

Although males did not display external evidence (e.g., scrotal testes [39]) of reproductive status, during each year of the study the population contained a subset of males that were noticeably smaller than most of the other males on the site. These smaller males were typically molting, resulting in a darker appearance due to the greater visibility of their black underfur, particularly along the neck and shoulders; based on their smaller size and distinctive pelage, we tentatively identified these individuals as subadults. To determine if the presence of conspicuous dark underfur was associated with a significant difference in body mass, we again used a linear model based on a Poisson distribution to evaluate the relationship between mass and putative age. Molting pelage was included as a fixed effect, with year included as a random effect. As with females, males captured during successive field seasons were assumed to be reproductive during their second year and hence the model was also run using data from second-year and molting males captured during the same field season.

### Demographic monitoring

Based on trapping records, we estimated values for several demographic parameters for each year of the study. Population density (adults/ha) was calculated by dividing the total number of adults resident on the study site by the size of the site; the number of adults used in these calculations included marked individuals as well as the estimated number of unmarked animals on the site. The sex ratio of the population was calculated for adults and for subadults using data from the animals captured. Age structure was examined by calculating the proportion of subadults in the study population, with relative age determined as described above. Between years, recapture rates were calculated by determining the proportion of individuals caught in a given year that were present on the study site during the following year; to generate a fourth pre-flood year of data, recapture rates from 2009 to 2010 were quantified using data on the composition of the study population in 2009 provided in [35]. Although these analyses allowed us to evaluate the tendency for different subsets of individuals (e.g., males versus females) to remain in the study population across multiple years, they did not allow us to distinguish loss of animals due to emigration versus mortality. As a result, we refer to these data simply as recapture rates.

### Quantifying spatial relationships

We used scan sampling of animal locations to characterize spatial relationships among members of the study population. At the start of each field season, a grid (8 m x 8 m cell size) was established on the study site using surveyors’ flags; the grid was labeled with a Cartesian coordinate system that allowed the location of an animal to be estimated by researchers located at pre-determined locations around the periphery of the study site. Localities recorded for small objects placed at known locations revealed this procedure to be accurate to within 1 m. To enhance the precision of our spatial data, the study site was divided into four non-overlapping sections, each of which was scanned by a different researcher. Visual scans were completed multiple times per day between sunrise and sunset (0700–2000 hrs), with at least one hour allowed between successive scans; at each time point, the four sections of the study site were scanned simultaneously. During a scan, the locality of each animal visible on the study site was recorded to the nearest meter. Previous research has shown that members of the study population are diurnal, with animals typically spending the night in subterranean nests located within their daytime home ranges [35]. As a result, our inability to conduct visual scans at night should not have biased our analyses of spatial relationships. Further, comparisons of home ranges based on scan sampling versus radio telemetry data revealed no significant differences in home range size [35], indicating that visual determination of animal locations provided a robust basis for characterizing spatial relationships among members of the study population.

Individual home ranges for adults in the study population were characterized using 95% Minimum Convex Polygons (MCPs), as implemented in the adehabitatHR package in R [41]. Detailed descriptions of these analyses are provided in [36]. In brief, home ranges were constructed for all animals for which at least six visual records were available. This exceeds the minimum number of data points required by adehabitatHR to construct a home range [41]; in all years of the study, the majority (> 90%) of the animals included in these analyses were characterized by > 10 observations (mean = 39.5 ± 22.4 observations per individual, range = 6–120, n = 184 animals over 5 years). Estimates of home range size were generated by adehabitatHR based on individual 95% MCPs; given the marked between-year variation in home range size and location reported for members of the study population [36], annual home ranges for individuals monitored during more than one field season were treated as independent.

Percent overlap of individual 95% MCPs was calculated using adehabitatHR; because overlap between pairs of individuals may not have been symmetric, overlap was calculated from the perspective of each individual in a pairwise comparison. To determine how much of the study site was occupied by tuco-tucos during each year of the study, we calculated the number of all grid cells on the site that were included in the home range of at least one member of the study population and then divided by the total number of grid cells monitored during that year. Although the boundaries of the site were generally consistent throughout the study, this procedure accounted for minor variations in the specific grid cells monitored during the different years of data collection.

### Quantifying social organization

To identify each social unit (i.e., spatially distinct subset of lone or group-living individuals) within the study population, data on home range overlap were analyzed using SOCPROG [42], as described by [35]. In brief, for each year of the study, pairwise values of percent home range overlap were used to create an association matrix that was then analyzed to identify statistically significant spatial clusters of individuals. Social units were identified as subsets of individuals that clustered together with cophenetic correlation coefficients that exceeded cutoff values generated by SOCPROG. Detailed descriptions of these analyses and the composition of each social unit identified are provided in [36]. For the purposes of this study, these analyses were used to determine annual values for the number of social units present on the study site as well as annual values for mean social unit size (mean number of adults).

### Statistical analyses

To assess the effects of the flood on the demography and behavior of the study population, we designated field seasons as either flood or non-flood years. The 2013 field season was the first to occur after the flood and coincided with the first post-flood spring breeding season for the study population. As a result, 2013 was identified as a flood year; this was the only field season to be given this designation. The remaining years of the study (2010, 2011, 2012, 2014) were identified as non-flood years. This included the three field seasons that occurred prior to the flood as well as the final year of the study, which occurred nearly two years after the flood.

Two general statistical approaches were employed. For variables for which only a single measure per year was available but data could be represented as one of two outcomes (annual recapture rates, sex ratios), Fisher’s Exact Tests were used to determine if values for 2013 differed from those from other years of the study. For variables for which multiple measures per year were available (home range size, social unit size, percent overlap of home ranges), standard two-sample or multi-sample tests were conducted using online calculators provided by Social Science Statistics (https://socscistatistics.com). Because our data typically violated assumptions of normality and equality of variances, non-parametric analyses were used. All p-values reported are two-tailed unless indicated otherwise.

## Results

Two hundred and eight highland tuco-tucos (84 males, 124 females) were captured on the study site from 2010 to 2014. Observations of unmarked individuals suggested that 13 animals resident on the site evaded capture during the course of the study (S1 Table). Within years, the consistent locations at which these animals were detected combined with field notes regarding individually distinctive pelage markings (e.g., molt patterns) provided a clear means of distinguishing between different uncaught animals. Based on these data, 2.6 ± 1.3 animals per year (range = 1–4, n = 5 years) evaded captured; accordingly, approximately 92.4 ± 5.5% of the animals present on the site during each year of the study were included in our analyses.

### Age composition of the study population

**Fig 2:**
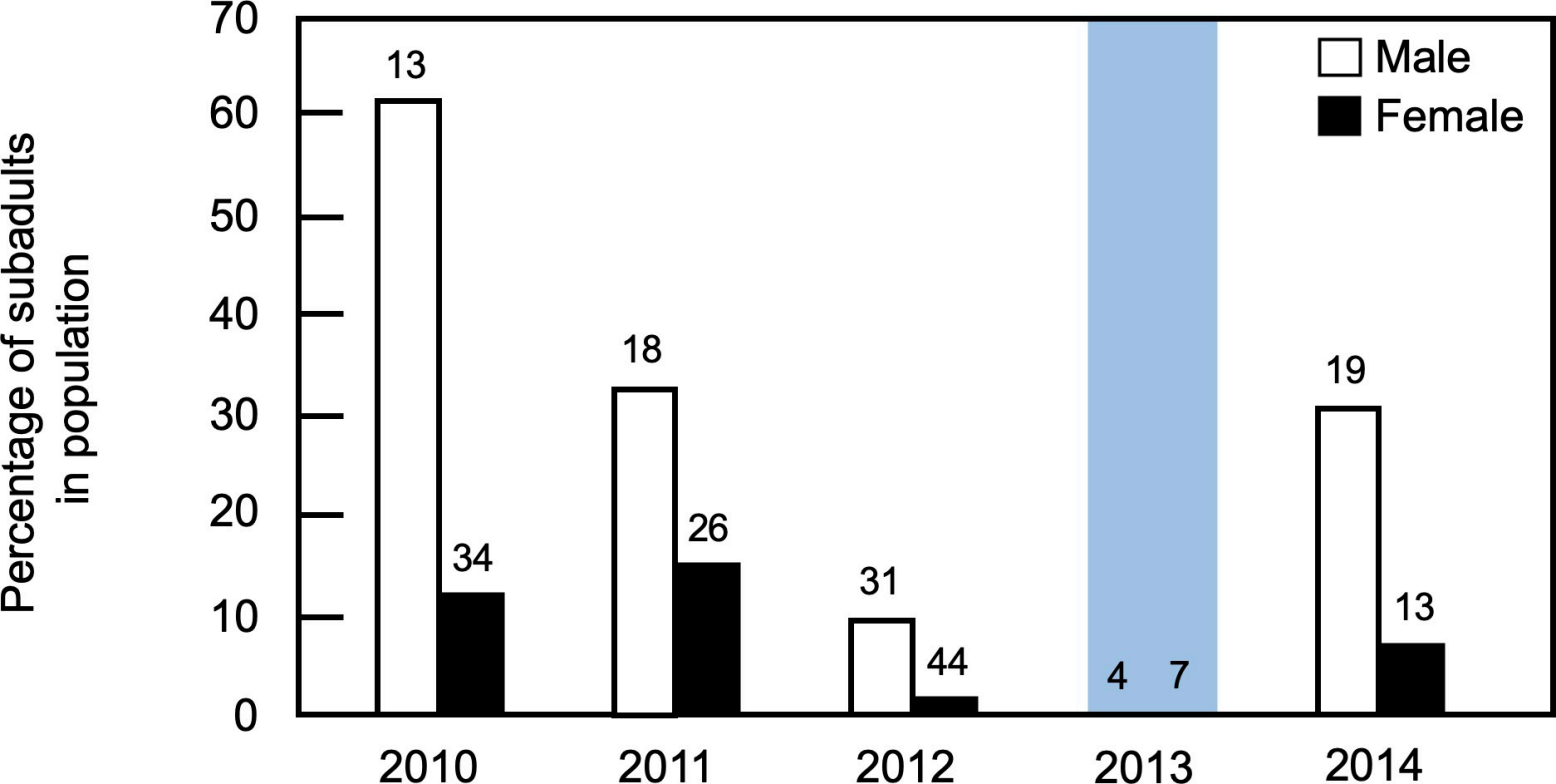
Age composition of the study population. The percentages of males and females in the study population identified as subadults are shown for each year of the study. The total number of animals (subadults and adults) of each sex is indicated for each year of data collection. The blue rectangle denotes data from the first post-flood field season.

In all years, the majority of females captured (93.4 ± 5.3% per year, range = 88.2–100.0%; n = 124 females over 5 years) were reproductively active. Coupled with observations of pre-weaned and recently weaned juveniles on the study site, these data suggest that November-December corresponds to the breeding season for the study population. Reproductive status was a significant predictor of female body mass, with masses for breeding animals being greater than those for non-breeders (effect size ± SE = -110.91 ± 15.82 g; t = 7.01, p = < 0.0001; S2 Table); year of capture did not affect this outcome (p = 0.09). Similarly, masses for recaptured (second-year) animals were significantly greater than those for non-breeders (effect size ± SE = 131.54 ± 13.96 g; t = 9.42 p < 0.0001; S2 Table); again, year of capture did not affect this outcome (p = 0.06). In each field season for which sufficient data were available, body masses for non-breeding females fell outside of 95% confidence intervals for both recaptured females and all breeding females, providing further evidence that non-breeding individuals were smaller animals that were likely to be subadults (S2 Table). Based on these criteria, the mean proportion of females per year identified as subadults was 5.3 ± 4.5% (range = 0.0–10.3%, n = 5 years; Fig 2; S3 Table).

Among males, the presence of conspicuous dark underfur was a significant predictor of body mass, with darker individuals being smaller than other males (effect ± SE = -140.84 ± 9.73 g; t-test = 14.46, p < 0.001; S2 Table); year of capture did not affect this outcome (p = 0.42). Body masses for recaptured (second-year) animals were significantly greater than those for males with darker, molting pelage (effect + SE = 144.74 ± 12.92 g; t-test = 11.20, p < 0.001; S2 Table); year of capture did not affect this outcome (p = 0.11). In each field season for which sufficient data were available, body masses for darker males fell outside of 95% confidence intervals for both recaptured males and all males lacking dark pelage, suggesting that smaller, darker individuals were likely subadults (S2 Table). Based on these criteria, the mean proportion of males per year identified as subadults was 27.2 ± 23.94% (range = 0.0–61.5%, n = 5 years; Fig 2; S3 Table).

### Effects of flood on adult population density

**Fig 3:**
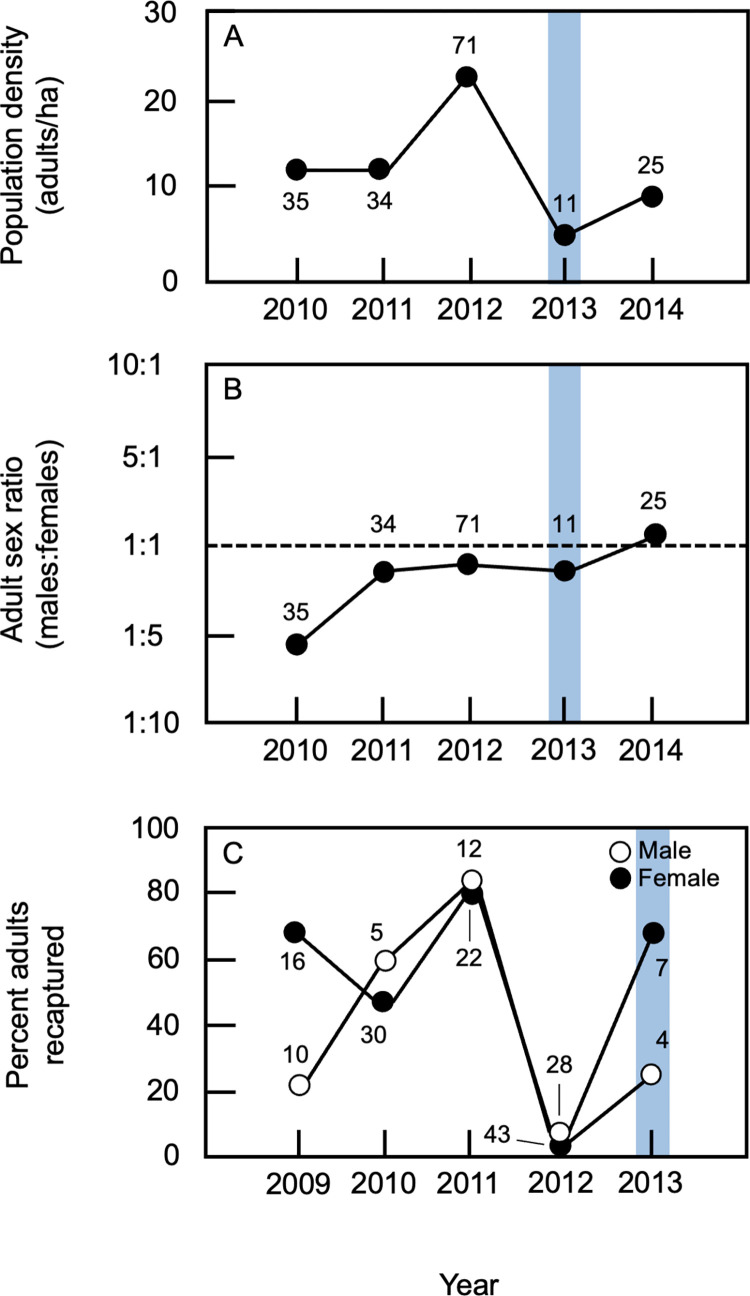
Adult composition of the study population. (A) Estimates of population density (number of adults per hectare) are shown for each year of the study. (B) The ratio of adult males to adult females is shown for each year. In both panels, numbers denote the total number of adults resident on the study site during each year of data collection. (C) Recapture rates for adult males and females; rates represent the percentage of adults captured in one year that were recaptured during the following year. Data for males and females are shown separately; numbers indicate the total number of adult males or females captured during a given year. Data from 2009 were obtained from [35]. In all panels, blue rectangles indicate data from the first post-flood field season.

Flooding of the Río Cincel in December 2012 had profound effects on the study population. During the following field season (November-December 2013), the mummified corpses of 10 tuco-tucos were found on the surface of the study site (Fig 1), suggesting that these individuals had been caught in water from the river, drowned, and their bodies left above ground to dry when the flood waters receded. Scanning of these corpses revealed that 6 (60.0%) still contained a PIT tag, confirming that those individuals had been members of the 2012 study population. Consistent with this scenario, population density during the 2013 field season (3.6 adults/ha) was ~ 75% less than the mean density (15.9 ± 7.6 adults/ha, range = 11.3–24.6 adults/ha, n = 3 years; Fig 3A, S4 Table) during pre-flood years. Although the limited number of field seasons for which data were available precluded statistical analysis of this difference, population density in 2013 fell outside the 95% confidence interval for pre-flood years (7.3–24.5 adults/ha), suggesting that this decrease was significant. By the 2014 field season, the density of the study population had increased to 9.3 adults/ha; this value was within the 95% confidence interval for pre-flood years.

### Effects of flood on adult recaptures

The sex ratio for adults in the study population was typically female-biased (Fig 3B; S5 Table); the sole exception to this pattern occurred during 2014, when adult males outnumbered adult females (1.0:0.9). For non-flood years, the mean percentage of animals (both sexes) recaptured during the following season was 58.1 ± 16.5% (n = 4 years) compared to a recapture rate of 2.8% from 2012 to 2013 (first post-flood year); this difference between recaptures in flood and non-flood years was significant (Fisher’s exact test, p < 0.0001; S5 Table). When recapture rates for non-flood years (n = 4) were examined as a function of sex, the mean percentage of recaptures for adult females (68.8 ± 16.3%) was greater than that for adult males (47.1 ± 30.0%; Fig 3C, S5 Table). For both sexes, the percentage of recaptures was < 4.0% from 2012 to 2013 (first post-flood year); for both males and females, this difference between recaptures in flood and non-flood years was significant (Fisher’s exact tests, both p < 0.001). Indeed, of the 43 adult females and 28 adult males captured during the 2012 field season, only one animal of each sex was recaptured during the 2013 (post-flood) season. These data indicate that the decrease in population density detected following the flood was associated with a pronounced decline in recaptures of marked adults relative to other years of the study, with this effect being particularly pronounced for females.

### Effects of flood on subadult recaptures

**Fig 4:**
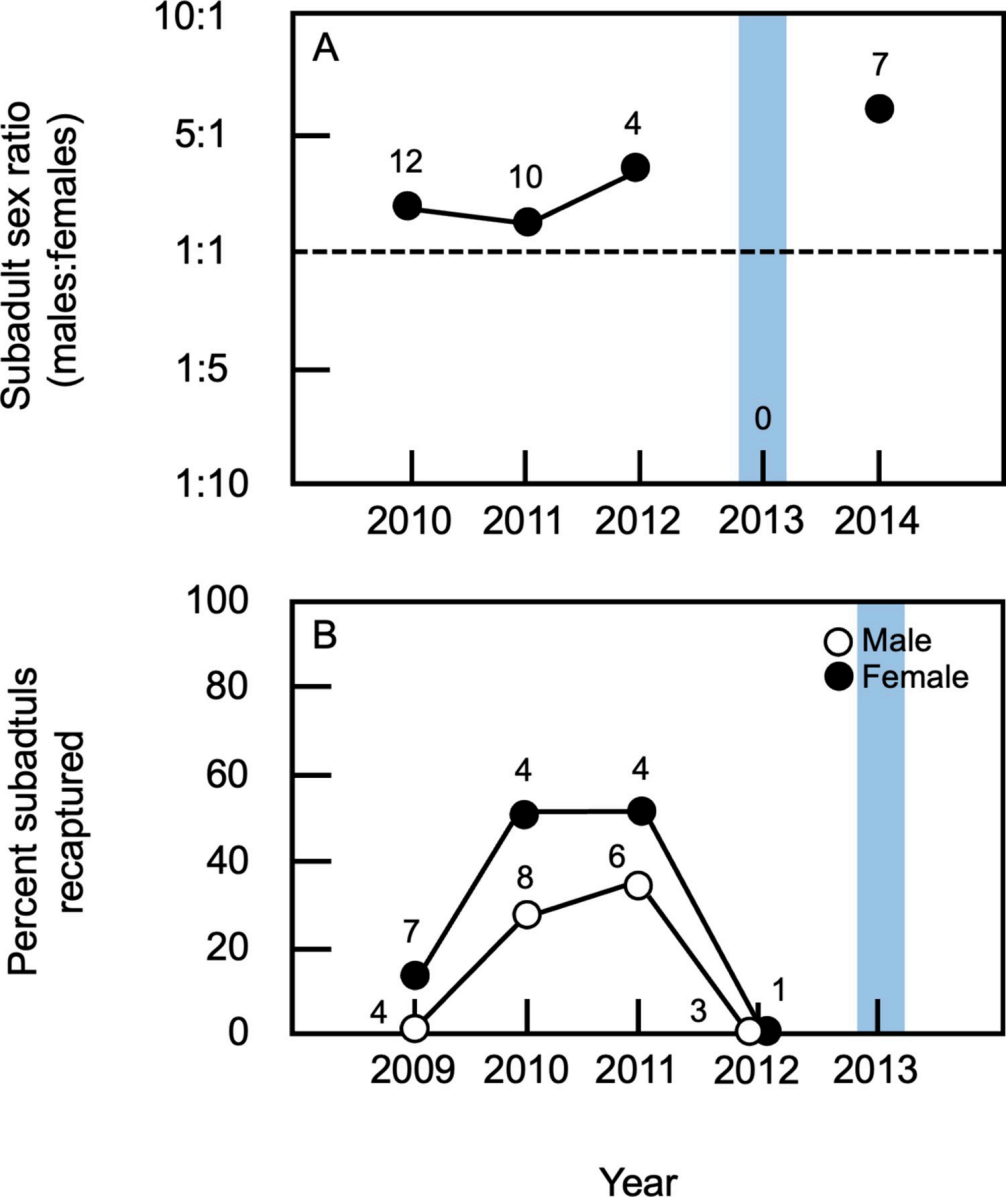
Subadult composition of the study population. (A) Ratio of subadult males to subadult females during each year of the study. The total number of subadults captured is indicated for each year of the study. Values above the dashed line denote male-biased sex ratios. (B) Recapture rates for subadult males and females; rates represent the percentage of subadults captured in one year that were recaptured during the following year. Data for males and females are shown separately; numbers indicate the total number of subadults of each sex captured during a given year. Data from 2009 were obtained from [35]. No subadults were present in 2013 and thus recapture rates could not be calculated for 2014. In all panels, blue rectangles indicate data from the first post-flood field season.

Given the limited number of adults recaptured in 2013, it is possible that the flood affected the age structure of the study population. In non-flood years, the mean percentage of animals in the study population that were subadults was 18.9 ± 9.2% (n = 4 years) when data for both sexes were considered (S3 Table). In contrast, no subadults were present in the population during the 2013 field season (Fig 2), indicating that the decrease in adult population density during this year was not associated with a relative increase in the percentage of subadults on the study site. In contrast to adults, when the proportion of subadults in the study population was examined as a function of sex, there was a clear tendency for subadult males to be more common than subadult females (Fig 2). Consistent with this, the sex ratio for subadults was male biased during all years when subadults were captured in the study population (Fig 4A, S6 Table). This difference in sex ratios between adults and subadults was significant (Fishers exact test, p = 0.0007).

Across all years of the study, the percentage of individuals (both sexes) captured as subadults in one year that were recaptured on the study site as adults during the following year ranged from 0.0% to 40.0% (mean = 28.6 ± 14.3%, n = 3 years; Fig 4B, S6 Table). For non-flood years, the mean percentage of subadult females recaptured in the following season was 38.1 ± 20.6% (n = 3 years) while the mean percentage for subadult males was 19.4 ± 17.3% (S6 Table). In contrast, no subadults captured in 2012 were recaptured in 2013 (Fig 2). Further, because no subadults were captured in the study population in 2013, no recaptures were possible in 2014. Although the limited number of subadults captured per year (n = 0–12) precluded statistical comparisons of these values, the flood appeared to result in the complete loss of subadults in the population from 2012 to 2013 and an absence of subadults in the population in 2013.

### Effects of flood on space use

Animal locations recorded during scan sampling were used to generate maps for 185 distinct home ranges (95% MCPs), of which 157 (85.9%) were occupied by adults. This included 50 home ranges for adult males (n = 44 animals) and 107 home ranges for adult females (n = 76 animals). During non-flood years, the mean percentage of grid cells on the study site (total n = 1000 cells) that were occupied (contained a portion of the home range for at least one animal) was 28.1 ± 5.8% (n = 4 years). In contrast, 25.4% of grid cells were occupied during 2013; this value fell within the 95% confidence interval for values from non-flood years (47.8%, 74.8%), suggesting that the total area used by members of the study population during the first post-flood season did not differ from that used during non-flood years.

**Fig 5:**
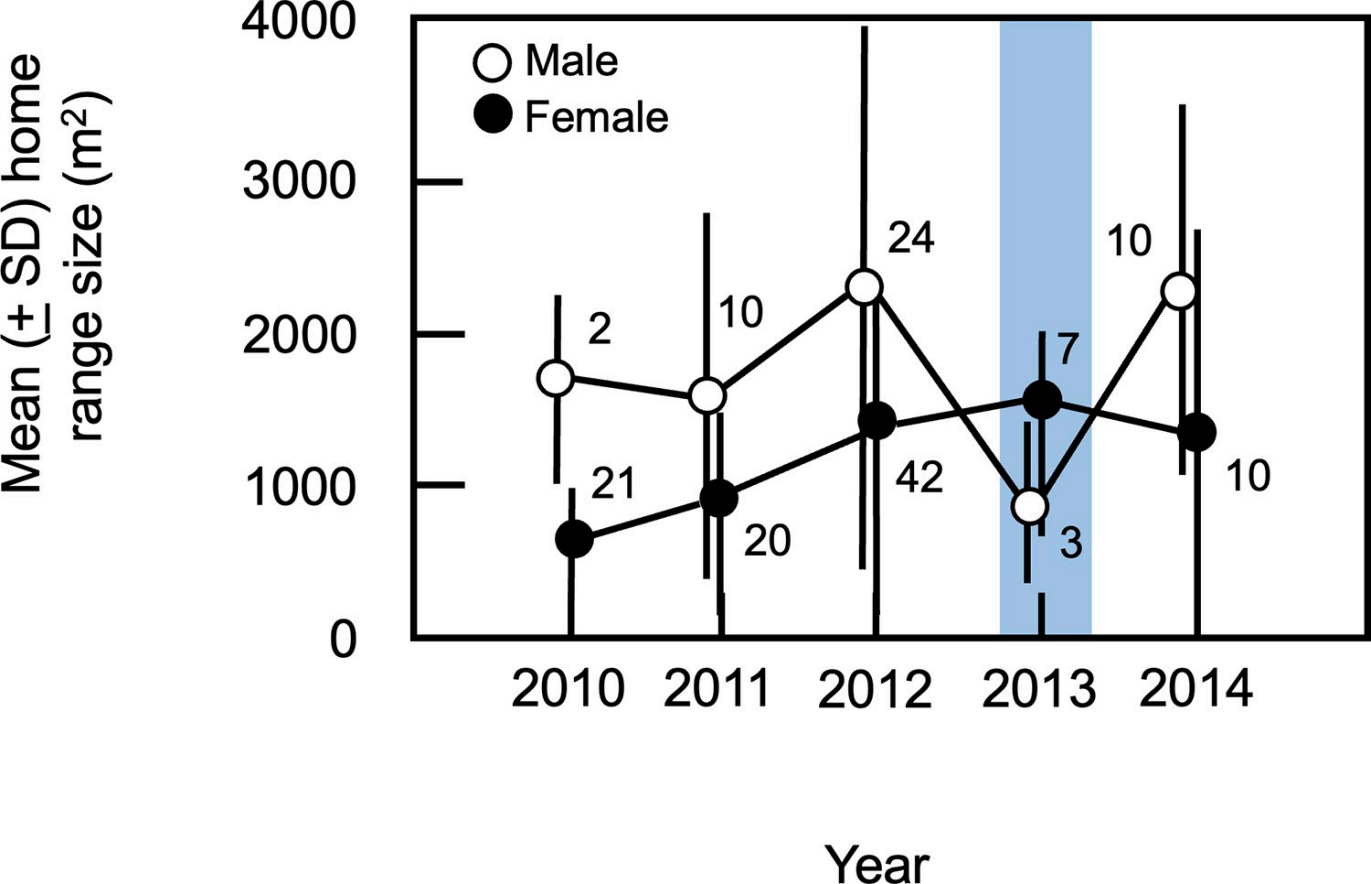
Mean (± 1 SD) home range sizes for adult males and females. The total number of home ranges measured for males and for females is indicated for each year of the study. The blue rectangle denotes data from the first post-flood field season.

Of the 157 home ranges occupied by adults, eight (5.1%) were excluded from subsequent analyses due to their extremely small sizes (< 100 m^2^; S7 Table). The mean number of observations used to generate each of these ranges was 12.8 ± 9.4 observations per individual compared to an overall mean of 39.5 ± 22.4 of fixes per animal, suggesting that a limited number of data points may have contributed to underestimation of the sizes of the home ranges that were excluded from analysis. Although mean home range size for adult males appeared to decline in 2013 (Fig 5; S7 Table), no significant variation in home range sizes was detected among years of the study (Kruskal-Wallis H = 5.67, df = 4, p = 0.225), likely due to the large variation in male home range sizes evident in most years. In contrast, home ranges sizes for adult females did vary among years (Kruskal-Wallis H = 12.05, df = 4, p = 0.014), although post-hoc comparisons revealed no significant pairwise contrasts between any years of the study (Mann-Whitney U tests, all p > 0.0117, Bonferroni-corrected alpha = 0.005; Fig 5, S7 Table). Thus, overall, home range sizes did not appear to be affected by the flood.

### Effects of flood on social organization

**Fig 6:**
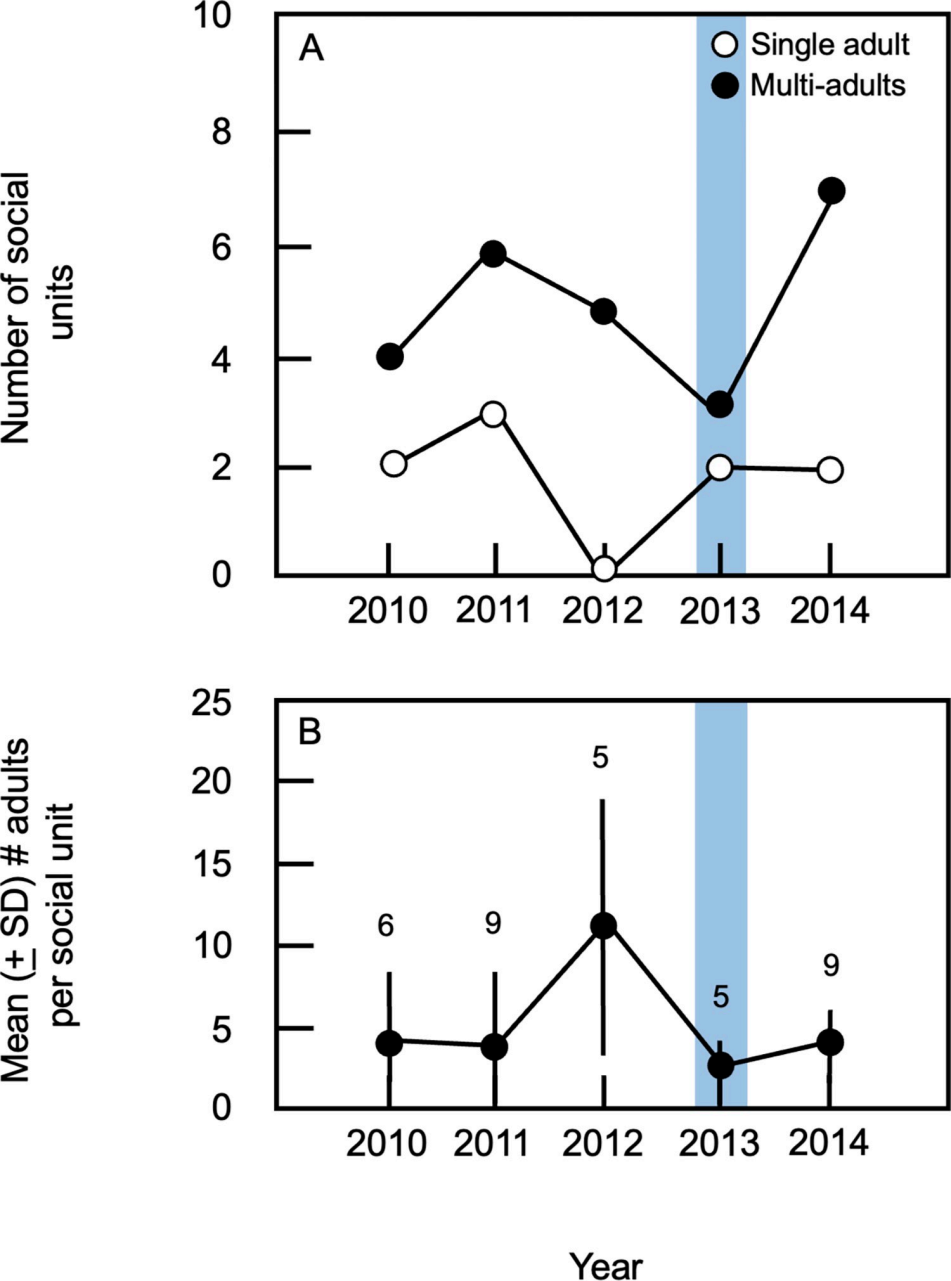
Summary of social units within the study population. (A) The number of social units present during each year of the study; data for single-adult versus multi-adult social units are shown separately. (B) The mean (± 1 SD) number of adults per social unit; numbers indicate the total number of social units present during a given year. In both panels, blue rectangles indicate data from the first post-flood field season.

Use of 95% MCPs to identify spatially distinct clusters of animals revealed that social units composed of multiple adults were common in the study population. The number of adults per social unit ranged from 1 to 23 (S8 Table); in all years, the number of social units containing multiple adults was greater than the number of lone individuals (Fig 6A). In non-flood years, the mean number of social units per field season was 7.3 ± 2.1 (range = 5–9, n = 4 years). In contrast, five social units were identified during the post-flood 2013 field season (Fig 6A); no adults assigned to the same social unit in 2013 had lived together in 2012. During non-flood years, the mean number of adults per social unit ranged from 2.3 (± 1.5) to 13.4 (± 7.1), with the number of adults per unit being greatest in 2012; in contrast, the mean number of adults per social unit in 2013 was 2.0 ± 1.2 (n = 5 social units; Fig 6B; S8 Table). When all years were considered, the number of adults per social unit varied significantly among years (Kruskal-Wallis test, H = 12.0, df = 4, p = 0.015). Post-hoc tests revealed that this outcome was due to significant contrasts between 2012 and all other years examined (Mann-Whitney U tests, all p < 0.023); no other significant pairwise differences between years were detected (Mann-Whitney U tests, all p > 0.197). Although mean social unit size was lower in 2013 than in 2012, three of the five social units on the study site during the 2013 field season contained multiple adults (Fig 6A) indicating that even during the year when adult population density was lowest, members of the study population continued to live together in groups.

**Fig 7:**
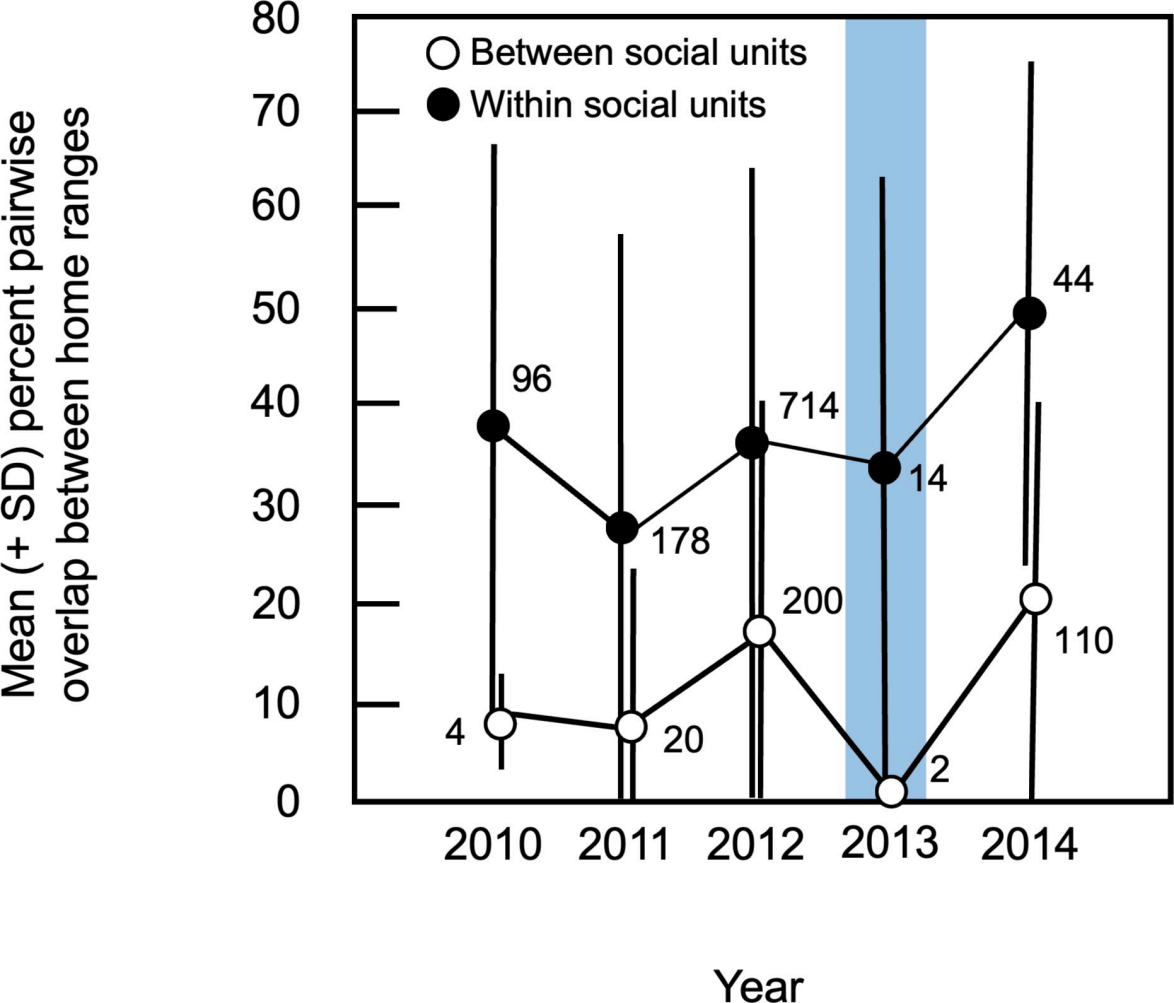
Mean (± 1 SD) percent pairwise overlap between individual home ranges. Values for overlap between members of the same (within) versus different (between) social units are shown separately; the number of pairs of overlapping animals used to calculate each mean is indicated. The blue rectangle indicates data from the first post-flood field season.

**Fig 8:**
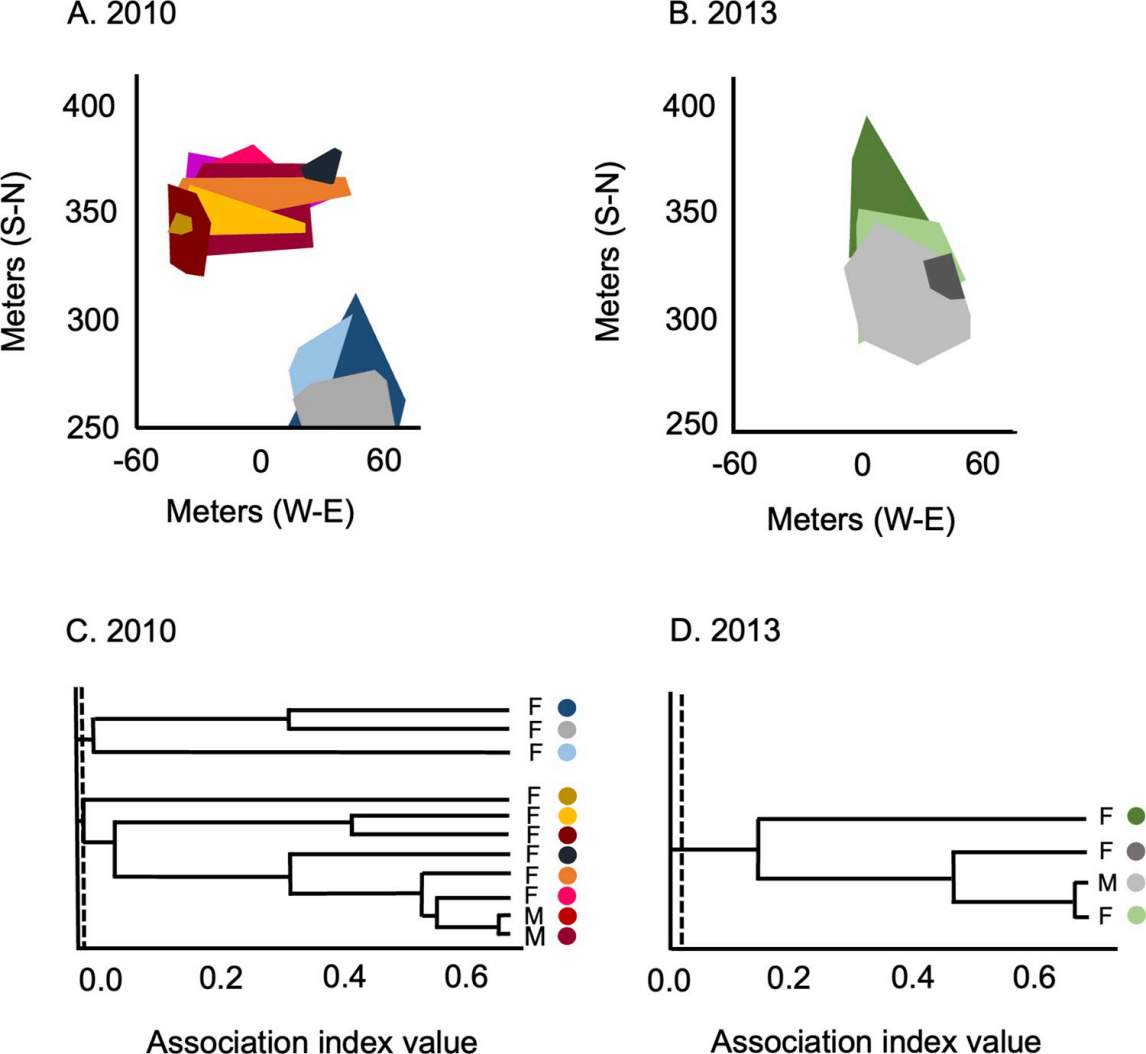
Comparison of spatial relationships among members of the study population before and after the flood. Minimum convex polygons (95%) are shown for adults present on the study site in (A) 2010 and (B) 2013. For both years, data are from the same ca. 1.5 ha portion of the study site. Dendrograms used to identify social units are shown in (C) and (D); two social units were present in 2010 versus only a single social unit in 2013. Dendrograms are based on association index values for these animals calculated from estimates of home range overlap; in both panels, the dashed vertical line denotes the cutoff index value for including animals in the same social unit. Colors used to distinguish polygons are the same as those used to identify individuals in the associated dendrogram.

Within social units, pairwise percent overlap of individual home ranges varied significantly among years (Kruskal-Wallis H = 27.03, df = 4, p < 0.0001; Fig 7, S9 Table). Post-hoc analyses revealed significant contrasts between percent overlap during 2014 and all other years of the study, with overlap being greater in 2014 (Mann-Whitney U tests, contrasts with 2014, all p < 0.014). In contrast, no significant differences were detected between 2013 and other years of the study (Mann-Whitney U tests, all p > 0.289), providing no evidence that spatial relationships within social units differed during the first post-flood field season. For spatial overlap between individuals assigned to different social units, small sample sizes in 2010 and 2013 precluded formal statistical analyses of these data (Fig 7, S9 Table). Mean percent overlap between members of different social units was lowest in 2013; the mean value for 2013 fell outside of 95% confidence intervals for all other years of the study, suggesting that this difference was significant (Fig 7; S9 Table). Thus, although distinct social units appeared to have been more widely distributed across the study site during the first post-flood year, spatial relationships among individuals within the same social unit were not affected by the flood (Fig 8).

## Discussion

Our analyses indicate that flooding by the Río Cincel in December 2012 had pronounced effects on the demography of the population of *C*. *opimus* located along the western bank of the river. During the first field season following the flood, the density of adults on the study site was markedly lower than in other years. This reduction in density was associated with a significant decrease in adult recaptures from 2012 to 2013 as well as the absence of subadults from the study population during the 2013 field season. Despite these demographic changes, tuco-tucos resident on the study site in 2013 continued to live in multi-adult social units. Neither the sizes of individual home ranges nor the percentage of the study site occupied by *C*. *opimus* increased after the flood, indicating that the observed decrease in density was not associated with an expansion of the area(s) occupied by members of the post-flood study population. Within social units, overlap of individual home ranges did not differ following the flood; among social units, overlap was reduced in 2013, indicating that groups tended to be more spatially distinct at low population density. Collectively, these findings suggest that group living in the study population does not occur due to constraints imposed by the presence of conspecifics.

### Demographic consequences of flood

Flooding of the study site appeared to result in substantial mortality among members of the study population. During the first post-flood field season, the density of adults on the study site was reduced by ~ 75% compared to the mean density for the other years monitored. This decline was associated with a significant decrease in the percentage of animals–adults and subadults–recaptured from the previous year. Although our data did not allow us to distinguish between individuals that died versus those that left the study site, the observed decline in recapture rates coupled with the lack of a significant increase in recruitment of unmarked individuals suggests that the flood was associated with substantial mortality of animals living on and in the vicinity of the site. This interpretation is supported by visual observations of numerous mummified corpses on the site during 2013; many of these remains contained PIT tags, confirming that they had been members of the study population during the 2012 field season. By 2014, the percentage of subadults in the population had returned to pre-flood levels, as had recapture rates for adults. Thus, while the effects of the flood appear to have been limited primarily to the 2013 field season, this event had profound short-term consequences for the density and demographic structure of the study population.

### Spatial consequences of flood

Changes in population density may affect spatial relationships among conspecifics in multiple ways. In general, such effects are expected to result from changes in home range size and overlap. For example, a reduction in density may allow remaining individuals to spread out within the habitat [20, 43]; if home range sizes remain the same, this should lead to a reduction in spatial overlap among conspecifics. Alternatively, reducing density may allow individuals to increase the sizes of their ranges [23, 44], with the result that overlap among conspecifics remains the same even though animals are more widely distributed across the habitat. In contrast to these scenarios, however, members of our study population displayed few changes in patterns of space use following the flood. We found no evidence that either the total area occupied by the population or the mean size of individual home ranges differed post-flood, indicating that the animals did not expand their individual or collective use of the habitat at low population density. Thus, overall, patterns of space use during the first post-flood year were not markedly different from those during non-flood years. This lack of change suggests that spatial relationships within the study population–particularly the size and placement of individual home ranges–are not driven by population density.

### Implications for social organization

Despite the pronounced reduction in population density from 2012 to 2013, multi-adult social units were present on our study site during the first post-flood field season. Animals assigned to the same social unit in 2013 had not lived together in 2012, indicating that the occurrence of multi-adult groups during the post-flood year did not reflect pre-flood spatial and social relationships. Mean social unit size in 2013 did not differ from that in most other years of the study, indicating that the persistence of multi-adult groups after the flood was not associated with unusually small group sizes, as might be expected if social units present in 2013 were simply the surviving remnants of groups present before the flood. Together with the lack of significant post-flood changes in patterns of space use, these findings suggest that group living in the study population does not reflect constraints imposed by conspecifics; even at low density, members of the population continued to live together in multi-adult groups.

It is possible that multi-adult groups were present in 2013 because the behavior of highland tuco-tucos is not sufficiently flexible to have allowed changes in social organization between successive breeding seasons. Differences in social organization associated with short-term fluctuations in population density have been reported for other rodent species including prairie voles (*Microtus ochrogaster*: [45]), great gerbils (*Rhombomys opimus*: [46]), and African striped mice (*Rhabdomys pumilo*: [47]), leading to the suggestion that such social flexibility may have important adaptive consequences for species exposed to variable demographic or ecological conditions [48]. Long-term monitoring of the tuco-tucos at Pozuelos has revealed that spatial and social relationships within the study population are dynamic, with individuals frequently changing social units between years [35, 36]. O’Brien et al. [36] have reported that this variability is not due to persistent individual-level differences in behavior but instead appears to reflect responses to immediate ecological and demographic conditions. Collectively, these findings suggest that the presence of multi-adult groups in 2013 was not due simply to the inability of members of our study population to respond to differences in pre- versus post-flood conditions, implying that even at very low population density, conditions on the study site favored the formation of social groups. Other factors that may contribute to group living have not been examined for *C*. *opimus*. In particular, further research is required to evaluate the effects of potentially important ecological parameters (e.g., distributions of critical resources such as food or burrow systems [1, 49, 50]) on the tendency for these animals to live in groups.

### Adaptive bases for group living

Whether groups form due to constraints imposed on living alone or due to benefits accrued by aggregating in the habitat can be viewed as different outcomes of the same cost-benefit equation [51]. These contrasting perspectives, however, have distinct implications for the fitness consequences of living alone versus within a group that may serve to guide thinking about the adaptive bases for group living. The constraints argument implies that individuals accrue greater direct fitness by living alone, with fitness being relatively lower when forced to live in groups [52, 53]. In contrast, the aggregation argument asserts that individuals co-occur spatially because the benefits of doing so exceed those associated with living alone elsewhere in the habitat [54, 55]. The fitness consequences of living alone versus within a group have not been quantified for *C*. *opimus*. In *C*. *sociabilis*–the only other group-living tuco-tuco studied to date–per capita annual direct fitness is greater for lone females [53], as predicted if individuals live together due to constraints imposed by the habitat. However, the social organizations of highland and colonial tuco-tucos differ with respect to several key features, as do the habitats in which these animals occur [56–59], suggesting that the fitness consequences of group living may also differ between these species. Studies that compare the direct fitness of lone versus group-living individuals and that evaluate the effects of group size on direct fitness are needed to understand the adaptive consequences of sociality in the Pozuelos population and to place data from *C*. *opimus* within the larger, comparative context of adaptive variation in mammalian social behavior.

## Conclusions

The abrupt, flood-induced decline in the density of the population of *C*. *opimus* located on the western bank of the Río Cincel provided an unexpected but important opportunity to explore interactions between demography and spatial and social relationships in this species of burrow-dwelling rodent. Despite a decrease in population density of ~ 75%, spatial and social relationships within this population remained largely unchanged and individuals continued to live in multi-adult social units during the first post-flood breeding season, suggesting that group living in these animals does not arise due to constraints imposed by the presence of conspecifics. Based on the outcome of this unplanned natural experiment, future efforts to understand the reasons for group living in highland tuco-tucos will focus on ecological factors such as the distribution of food and other critical resources. More generally, comparisons of pre- and post-flood data underscore the utility of catastrophic natural events for quantifying the adaptive bases for behavioral variation in natural populations of organisms.

## Supporting information

S1 Table Percentage of animals on the study site that were not caught. For each year of the study, visual sightings of unmarked animals were used to estimate the number of individuals that evaded capture. This number is given, as is the percentage of all animals detected on the study site each year that were uncaught.
(PDF)

S2 Table Mean (± 1 SD) body mass for members of the study population. For each year of the study, mean body mass is shown for (A) females and (B) males. For females, values shown are for all individuals, for animals that had been captured during the previous season (i.e., were known to be adults), and for individuals that were non-reproductive at the time of capture. For males, values are shown for all individuals, for animals that had been captured during the previous season (i.e., were known to be adults), and for a subset of individuals that were distinguished by extensive molting of pelage at the time of capture. N represents the number of individuals included in the calculation of each mean value.
(PDF)

S3 Table The percentage of subadults in the study population during each year of the study. The percentage of animals identified as subadults are shown for (A) both sexes and (B) males versus females. Subadults were identified based on body mass and either reproductive status (females) or pelage attributes (males).
(PDF)

S4 Table Estimates of population density for each year of the study. The total number of adults on the study site (captured and uncaught animals) was divided by the size of the site to generate annual estimates of density.
(PDF)

S5 Table Adult composition of the study population during each year of the study. In (A), data on adult sex ratios are shown. In (B), the percentage of animals captured in one year that were recaptured during the following year is shown for males, females, and all adults combined. Data on animals present in the study population during 2009 are from [35]. Because the study ended in 2014, no data are available regarding animals still resident in the study population in 2015.
(PDF)

S6 Table Subadult composition of the study population during each year of the study. In (A), subadult sex ratios are shown for each year of the study. In (B), the percentage of subadults captured in a given year that were recaptured in the following year is shown for males, females, and for both sexes combined. Data on composition of the study population in 2009 are from [35]. Because the study ended in 2014, no data are available regarding animals still resident in the study population in 2015.
(PDF)

S7 Table Home range sizes (m^2^) for adults in the study population. Estimates of individual home range sizes are based on 95% minimum convex polygons (MCPs) constructed from radiotelemetry data obtained from members of the study population. For each year, data for males and females are shown separately. For each year, the mean (± 1 SD) for each sex is given. N represents the number of individuals included in the calculation of each mean value. Values highlighted in blue were excluded from subsequent analyses as outliers.
(PDF)

S8 Table Social unit sizes for each year of the study. Social units were identified based on SOCPROG analyses of spatial overlap between 95% minimum convex polygons (MCPs) for adults residents on the study site. Values given represent the total number of adults per social unit.
(PDF)

S9 Table Mean percent overlap of individual home ranges. Mean (± 1 SD) pairwise values for percent overlap of 95% minimum convex polygons (MCPs) are given for animals assigned to (A) the same social unit and (B) different social units. Data for each year of the study are presented separately. Pairwise values for proportion of home range overlap (95% MCPs) between individuals are given for animals assigned to (C) the same social unit and (D) different social units. Because pairwise estimates of overlap were not symmetric, separate estimates of percent overlap were calculated from the perspective of each individual in an overlapping pair.
(PDF)
