# Supplementary material for: Group living in highland tuco-tucos (Ctenomys opimus) persists despite a catastrophic decline in population density
Source: PLoS One. 2024 Jun 7;19(6):e0304763. doi: 10.1371/journal.pone.0304763 (PMC11161065; doi:10.1371/journal.pone.0304763)
Supplement: S1 Table — For each year of the study, visual sightings of unmarked animals were used to estimate the number of individuals that evaded capture. This number is given, as is the percentage of all animals detected on the study site each year that were uncaught. (PDF) [file pone.0304763.s001.pdf]

Supplementary Table 1:

Percentage of animals on the study site that were not caught. For each year of the study, visual sightings of unmarked animals were used to estimate the number of individuals that evaded capture. This number is given, as is the percentage of all animals detected on the study site each year that were uncaught.

Year	# uncaught	Total # caught	% uncaught
2010	1	47	2.1
2011	2	44	4.3
2012	4	74	5.1
2013	2	11	15.4
2014	4	32	11.1
Mean	2.6	41.6	7.6
SD	1.3	23.0	5.5
