# Supplementary material for: Group living in highland tuco-tucos (Ctenomys opimus) persists despite a catastrophic decline in population density
Source: PLoS One. 2024 Jun 7;19(6):e0304763. doi: 10.1371/journal.pone.0304763 (PMC11161065; doi:10.1371/journal.pone.0304763)
Supplement: S2 Table — For each year of the study, mean body mass is shown for (A) females and (B) males. For females, values shown are for all individuals, for animals that had been captured during the previous season (i.e., were known to be adults), and for individuals that were non-reproductive at the time of capture. For males, values are shown for all individuals, for animals that had been captured during the previous season (i.e., were known to be adults), and for a subset of individuals that were distinguished by extensive molting of pelage at the time of capture. N represents the number of individuals included in the calculation of each mean value. (PDF) [file pone.0304763.s002.pdf]

Supplementary Table 2:

Mean (± 1 SD) body mass (g) for members of the study population. For each year of the study, mean body mass is shown for (A) females and (B) males. For females, values are shown for all individuals, for animals that had been captured during the previous season (i.e., were known to be adults), and for individuals that were non-reproductive at the time of capture. For males, values are shown for all individuals, for animals that had been captured during the previous season (i.e., were known to be adults), and for a subset of individuals that were distinguished by extensive molting of pelage at the time of capture. N represents the number of individuals included in the calculation of each mean value.

				Year		
A. Females			2010	2011	2012	2013	2014
Breeding							
	All	Mean	263.8	304.0	287.4	295.7	307.4
		SD	40.5	41.5	48.5	31.0	39.9
		N	30	22	43	7	12
		95% CI	(249, 278)	(287, 321)	(273, 302)	(273, 319)	(285, 330)
	Recaptures	Mean	280.0	313.5	315.6	350	324.8
		SD	18.7	30.6	43.1	NA	30.7
		N	11	13	18	1	5
		95% CI	(269, 291)	(297, 330)	(296, 336)	NA	(298, 352)
Non-breeding		Mean	201.3	205.7	155.0	NA	120.0
		SD	27.8	51.8	NA	NA	NA
		N	4	4	1	0	1

B. Males		Year				
		2010	2011	2012	2013	2014
Breeding						
All	Mean	362.0	340.4	365.6	381.3	387.7
	SD	55.1	34.8	26.8	58.4	36.1
	N	5	12	28	4	13
	95% CI	(314, 410)	(321, 360)	(356, 376)	(324, 439)	(368, 407)
Recaptures	Mean	324.8	364.7	380.0	345.0	440.0
	SD	30.7	8.4	24.2	NA	NA
	N	2	3	10	1	1
	95% CI	(282, 367)	(355, 374)	(365, 395)	NA	NA
Molting pelage	Mean	263.1	234.7	215.0	NA	173.3
	SD	24.9	24.9	25.0	NA	41.8
	N	8	6	3	0	6
