# Supplementary material for: Group living in highland tuco-tucos (Ctenomys opimus) persists despite a catastrophic decline in population density
Source: PLoS One. 2024 Jun 7;19(6):e0304763. doi: 10.1371/journal.pone.0304763 (PMC11161065; doi:10.1371/journal.pone.0304763)
Supplement: S3 Table — The percentage of animals identified as subadults are shown for (A) both sexes and (B) males versus females. Subadults were identified based on body mass and either reproductive status (females) or pelage attributes (males). (PDF) [file pone.0304763.s003.pdf]

Supplementary Table 3:

The percentage of subadults in the study population during each year of the study. The percentage of animals identified as subadults are shown for (A) both sexes and (B) males versus females. Subadults were identified based on body weight and either reproductive status (females) or pelage attributes (males).

A. Percentage of subadults in study population

Year	# subadult males	# subadult females	Total # subadults	# adults	Total # animals	% subadults
2010	8	4	12	35	47	25.5
2011	6	4	10	34	44	22.7
2012	3	1	4	71	75	5.3
2013	0	0	0	11	11	0
2014	6	1	7	25	32	21.9

B. Percentage of subadults in study population by sex

Year	# subadult males	Total # males	% male subadults	# subadult females	Total # females	% female subadults
2010	8	13	61.5	4	34	11.8
2011	6	18	33.3	4	26	15.4
2012	3	31	9.7	1	44	2.3
2013	0	4	0.0	0	7	0.0
2014	6	19	31.6	1	13	7.7
