# Supplementary material for: Group living in highland tuco-tucos (Ctenomys opimus) persists despite a catastrophic decline in population density
Source: PLoS One. 2024 Jun 7;19(6):e0304763. doi: 10.1371/journal.pone.0304763 (PMC11161065; doi:10.1371/journal.pone.0304763)
Supplement: S4 Table — The total number of adults on the study site (captured and uncaught animals) was divided by the size of the site to generate annual estimates of density. (PDF) [file pone.0304763.s004.pdf]

Supplementary Table 4:

Estimates of population density for each year of the study. The total number of adults on the study site (captured and uncaught animals) was divided by the size of the site to generate annual estimates of density.

Year	Total # adults	Site area (ha)	Population density
2010	35	3	11.7
2011	34	3	11.3
2012	71	3	24.6
2013	11	3	3.6
2014	25	3	9.3
