# Supplementary material for: Group living in highland tuco-tucos (Ctenomys opimus) persists despite a catastrophic decline in population density
Source: PLoS One. 2024 Jun 7;19(6):e0304763. doi: 10.1371/journal.pone.0304763 (PMC11161065; doi:10.1371/journal.pone.0304763)
Supplement: S5 Table — In (A), data on adult sex ratios are shown. In (B), the percentage of animals captured in one year that were recaptured during the following year is shown for males, females, and all adults combined. Data on animals present in the study population during 2009 are from [35]. Because the study ended in 2014, no data are available regarding animals still resident in the study population in 2015. (PDF) [file pone.0304763.s005.pdf]

Supplementary Table 5:

Adult composition of the study population during each year of the study. In (A), data on adult sex ratios are shown. In (B), the percentage of animals capture in one year that were recaptured during the following year is shown for males, females, and all adults combined. Data on animals present in the study population during 2009 are from [35]. Because the study ended in 2014, no data are available regarding animals still resident in the study population in 2015.

A. Adult sex ratios

Year	# adult males	# adult females	Ratio M:F
2010	5	30	1 to 6
2011	12	22	1 to 1.8
2012	28	43	1 to 1.5
2013	4	7	1 to 1.8
2014	13	12	1 to 0.9

B. Adult recaptures

Year	# adult males	# recaptured next year	% recaptures	# adult females	# recaptured next year	% recaptures	# adults	# recaptured next year	% recaptures
2009	10	2	20.0	16	11	68.8	26	13	50
2010	5	3	60.0	30	13	43.3	35	16	45.7
2011	12	10	83.3	22	18	81.8	34	28	82.3
2012	28	1	3.6	43	1	2.3	71	2	2.8
2013	4	1	25.0	7	5	71.4	11	6	54.5
2014	13	NA	NA	12	NA	NA	25	NA	NA
