# Supplementary material for: Group living in highland tuco-tucos (Ctenomys opimus) persists despite a catastrophic decline in population density
Source: PLoS One. 2024 Jun 7;19(6):e0304763. doi: 10.1371/journal.pone.0304763 (PMC11161065; doi:10.1371/journal.pone.0304763)
Supplement: S6 Table — In (A), subadult sex ratios are shown for each year of the study. In (B), the percentage of subadults captured in a given year that were recaptured in the following year is shown for males, females, and for both sexes combined. Data on composition of the study population in 2009 are from [35]. Because the study ended in 2014, no data are available regarding animals still resident in the study population in 2015. (PDF) [file pone.0304763.s006.pdf]

**Supplementary Table 6:**

Subadult composition of the study population during each year of the study. In (A), subadult sex ratios are shown for each year of the study. In (B), the percentage of animals in the study population that were subadults are shown for males, females, and for both sexes combined. In (C ), the percentage of subadults captured in a given year that were recaptured in the following year is shown for males, females, and for both sexes combined. Data on composition of the study population in 2009 are from [35]. Because the study ended in 2014, no data are available regarding animals still resident in the study population in 2015.

A. Subadult sex ratios

| Year | # subadult males | # subadult females | Ratio M:F |
|---|---|---|---|
| 2010 | 8 | 4 | 2 to 1 |
| 2011 | 6 | 4 | 1.5 to 1 |
| 2012 | 3 | 1 | 3 to 1 |
| 2013 | 0 | 0 | NA |
| 2014 | 6 | 1 | 6 to 1 |

B. Subadult recaptures

| Year | # subadult males | # recaptured next year | % recaptures | # subadult females | # recaptured next year | % recaptures | Total # subadults | # recaptured next year | % recaptures |
|---|---|---|---|---|---|---|---|---|---|
| 2009 | 4 | 0 | 0.0 | 7 | 1 | 14.3 | 11 | 1 | 12.5 |
| 2010 | 8 | 2 | 25.0 | 4 | 2 | 50.0 | 12 | 4 | 33.3 |
| 2011 | 6 | 2 | 33.3 | 4 | 2 | 50.0 | 10 | 4 | 40.0 |
| 2012 | 3 | 0 | 0.0 | 1 | 0 | 0.0 | 4 | 0 | 0.0 |
| 2013 | 0 | NA | NA | 0 | NA | NA | 0 | NA | NA |
| 2014 | 6 | NA | NA | 1 | NA | NA | 7 | NA | NA |
