# Supplementary material for: Group living in highland tuco-tucos (Ctenomys opimus) persists despite a catastrophic decline in population density
Source: PLoS One. 2024 Jun 7;19(6):e0304763. doi: 10.1371/journal.pone.0304763 (PMC11161065; doi:10.1371/journal.pone.0304763)
Supplement: S7 Table — Estimates of individual home range sizes are based on 95% minimum convex polygons (MCPs) constructed from radiotelemetry data obtained from members of the study population. For each year, data for males and females are shown separately. For each year, the mean (± 1 SD) for each sex is given. N represents the number of individuals included in the calculation of each mean value. Values highlighted in blue were excluded from subsequent analyses as outliers. (PDF) [file pone.0304763.s007.pdf]

Supplementary Table 7:

Home ranges sizes (sq m) for adults in the study population. Estimates of individual home range sizes are based on 95% minimum convex polygons (MCPs) constructed from radiotelemetry data obtained from members of the study population. For each year, data for males and females are shown separately. For each year, the mean (± 1 SD) for each sex is given. N represents the number of individuals included in the calculation of each mean value. Values highlighted in blue were excluded from subsequent analyses as outliers.

	Year									
	2010		2011		2012		2013		2014	
	Males	Females	Males	Females	Males	Females	Males	Females	Males	Females
Home range size (sq m)	1182	2	178.5	97	3767	813	1307	1634	1459.5	960.5
	2033.5	186	522	226.5	2355.5	723	492	1298.5	1615	611.5
		353	2525	90	1982	1631.5	491.5	1461.5	2791.5	1476.5
		204	133.5	126	910.25	2185		235.5	2277.5	99.75
		1024.5	1481	475.5	1972.5	4018		962.25	1681.5	304.5
		77	2638	297.5	1443	963.25		2028.5	389	3573
		600	1927	443	1681	1035		2607	8	4077.5
		390.5	4043	733.5	7734.5	1791			3844	418
		512.75	467.5	548	906	447			4103.5	288
		1107	1333	783.5	3603.5	927			2155	371
		594		347.5	5216	1993			2085	
		39		168	4410	5637				
		535		1250	1301	638				
		1638		1251.5	400	919.5				
		655.5		2745.25	459.5	4348				
		511.5		2186	2234.5	929				
		430		237	2116	1793				
		291		670	1576.5	421				
		514		2787.5	1771.5	934				
		834		574.5	3475.75	708				
		959.5		1638	1829.5	1151.5				
		393.75		1125.5	1113.5	290				
		334.5			1006	1037				
		1250.5			537	524				

excluded from analysis

		59.5				1053.5				
						881				
						1126.5				
						471				
						327				
						543.5				
						1513.5				
						706				
						907				
						1594.5				
						815				
						2172				
						623.5				
						325				
						290.5				
						582				
						1525.5				
						234				
						70				
Mean	1607.8	637.5	1524.9	930.7	2241.8	1227.3	763.5	1461.0	2240.2	1218
SD	602.1	365.3	1276.0	822.5	1720.6	111.7	470.7	757.4	1111.6	1434.9
N	2	21	10	20	24	42	3	7	10	10
