# Supplementary material for: Group living in highland tuco-tucos (Ctenomys opimus) persists despite a catastrophic decline in population density
Source: PLoS One. 2024 Jun 7;19(6):e0304763. doi: 10.1371/journal.pone.0304763 (PMC11161065; doi:10.1371/journal.pone.0304763)
Supplement: S8 Table — Social units were identified based on SOCPROG analyses of spatial overlap between 95% minimum convex polygons (MCPs) for adults residents on the study site. Values given represent the total number of adults per social unit. (PDF) [file pone.0304763.s008.pdf]

Supplemental Table 8:

Social unit sizes for each year of the study. Social units were identified based on SOCPROG analyses of spatial overlap between 95% minimum convex polygons (MCPs) for adults resident on the study site. Values given represent the total number of adults per social unit.

	Year				
	2010	2011	2012	2013	2014
# adults per unit	1	1	9	1	1
	1	1	9	1	1
	7	3	23	2	2
	6	3	7	4	2
	3	10	19	2	2
	8	9			2
		1			3
		3			2
		1			6
N	6	9	5	5	9
Mean	4.3	3.6	13.4	2.0	2.3
St Dev	3.1	3.5	7.1	1.2	1.5
