# Supplementary material for: Group living in highland tuco-tucos (Ctenomys opimus) persists despite a catastrophic decline in population density
Source: PLoS One. 2024 Jun 7;19(6):e0304763. doi: 10.1371/journal.pone.0304763 (PMC11161065; doi:10.1371/journal.pone.0304763)
Supplement: S9 Table — Mean (± 1 SD) pairwise values for percent overlap of 95% minimum convex polygons (MCPs) are given for animals assigned to (A) the same social unit and (B) different social units. Data for each year of the study are presented separately. Pairwise values for proportion of home range overlap (95% MCPs) between individuals are given for animals assigned to (C) the same social unit and (D) different social units. Because pairwise estimates of overlap were not symmetric, separate estimates of percent overlap were calculated from the perspective of each individual in an overlapping pair. (PDF) [file pone.0304763.s009.pdf]

Supplementary Table 9.

Mean (+ 1 SD) pairwse values for percent overlap of 95% minimum convex polygons (MCPs) are given for animals assigned to (A) the same social unit and (B) different social units. Data for each year of the study are presented separately. Pairwise values for porportion of home range overlap (95% MCPs) between individuals are given for animals assigned to (C) the same social unit and (D) different social units. Because pairwse estimates of overlap were not symmetric, separate estimates of percent overlap were calculated from the perspective of each indvidual in an overlapping pair.

A. Within social units

		Year				
		2010	2011	2012	2013	2014
% pairwise overlap of 95% MCPs	Mean	37.2	28.1	36.6	33.4	48.7
	SD	30.6	20.1	30.3	30.5	26.5
	N	96	178	713	14	44

B. Between social units

		Year				
		2010	2011	2012	2013	2014
% pairwise overlap of 95% MCPs	Mean	8.0	8.6	18.8	1.3	20.9
	SD	4.0	15.0	21.2	1.3	20.9
	N	4	20	200	2	110
	95% CI	[illegible].08, 11.92)	(2.03, 15.17)	(17.11, 20.49)	(-0.50, 3.10)	(16.99, 24.81)

C. Within social units

	Year				
	2010	2011	2012	2013	2014
Proportion pairwise overlap of 95% MCPs	0.666	0.116	0.820	0.202	0.979
	0.387	0.333	0.017	0.069	0.197
	0.409	0.003	0.116	0.086	0.391
	0.011	0.050	0.760	0.268	0.895
	0.054	0.197	0.674	0.094	0.916
	0.013	0.015	0.921	0.759	0.193
	0.363	0.675	0.346	0.372	0.079
	0.029	0.433	0.498	0.171	0.522
	0.337	0.011	0.382	0.807	0.595
	0.127	0.200	1.000	0.921	0.799
	0.204	0.154	0.293	0.591	0.637
	0.471	0.007	0.656	0.096	0.248
	0.140	0.419	0.120	0.083	0.479
	0.090	0.088	0.427	0.150	0.236
	0.055	0.705	0.121		0.586
	0.077	0.054	0.604		0.605
	0.156	0.003	0.444	0.334	0.191
	0.146	0.008	0.051	0.305	0.740
	0.542	0.115	0.063	14	0.281
	0.555	0.038	0.658		0.417
	0.555	0.018	0.215		0.354
	0.150	0.750	0.951		0.322
	0.218	0.928	1.000		0.374
	0.084	0.959	0.180		0.547
	0.482	0.558	0.403		0.302
	0.003	0.484	0.002		0.923
	0.412	0.645	0.007		0.095
	0.741	0.520	0.445		0.540
	0.302	0.061	0.499		0.361
	0.032	0.043	0.151		0.669

0.818	0.304	0.342	0.731
0.362	0.455	0.000	0.231
0.071	0.000	0.102	0.987
0.207	0.108	0.517	0.707
0.292	0.499	0.325	0.369
0.086	0.034	0.260	0.884
0.023	0.596	0.210	0.935
0.907	0.919	0.882	0.212
0.218	0.648	0.141	0.187
0.062	0.570	0.178	0.455
0.159	0.986	0.335	0.192
0.703	0.945	0.391	0.374
0.479	0.697	0.894	0.395
0.633	0.800	0.417	0.284
0.097	0.334	0.783	
0.868	0.205	0.395	0.487
0.008	0.506	0.343	0.265
0.620	0.708	0.017	44
0.876	0.929	0.343	
0.233	0.930	0.141	
0.082	1.000	0.396	
0.132	0.188	0.810	
0.184	0.003	0.040	
0.593	0.054	0.023	
0.849	0.154	0.668	
0.564	0.434	0.967	
0.523	0.412	0.555	
0.856	0.087	0.148	
0.601	0.414	0.459	
0.070	0.302	0.408	
0.492	0.018	0.841	
0.060	0.125	0.791	
0.091	0.005	0.070	
0.812	0.038	0.508	

0.677	0.071	0.977
0.947	0.440	0.030
0.289	0.393	0.262
0.995	0.168	0.501
0.979	0.185	0.942
0.813	0.191	0.412
0.008	0.028	0.320
0.763	0.044	0.522
0.999	0.091	0.011
0.588	0.168	0.982
0.006	0.000	0.415
0.257	0.858	0.023
0.257	0.157	0.039
0.049	0.015	0.695
0.035	0.076	0.005
0.594	0.153	0.140
0.495	0.324	0.132
0.010	0.732	0.591
0.010	0.099	0.684
0.248	0.543	0.951
0.107	0.103	0.092
0.855	0.113	0.759
0.560	0.539	0.839
0.007	0.371	0.451
0.555	0.151	0.441
0.303	0.013	0.042
0.263	0.052	0.791
0.913	0.003	0.839
0.392	0.054	0.559
0.059	0.154	0.096
0.444	0.434	0.223
0.795	0.412	0.073
	0.087	0.995
	0.414	0.766

0.372	0.302	0.005
0.306	0.018	0.246
96.000	0.125	0.055
	0.005	0.022
	0.038	0.001
	0.071	0.581
	0.440	0.873
	0.393	0.170
	0.168	0.323
	0.185	0.489
	0.191	0.923
	0.028	0.155
	0.044	0.566
	0.091	0.842
	0.168	0.678
	0.000	0.358
	0.858	0.039
	0.157	0.700
	0.015	0.322
	0.076	0.204
	0.153	0.451
	0.324	0.808
	0.732	0.033
	0.099	0.377
	0.543	0.732
	0.103	0.695
	0.113	1.000
	0.539	0.988
	0.371	0.054
	0.151	0.245
	0.013	0.785
	0.052	1.000
	0.017	0.005
	0.041	0.023

0.113	0.131
0.900	0.164
0.012	0.026
0.377	0.071
0.021	0.056
0.092	0.046
0.043	0.366
0.077	0.555
0.009	0.503
0.070	0.161
0.466	0.062
0.994	0.013
0.021	0.532
0.836	0.399
0.045	0.076
0.279	0.094
0.015	0.141
0.171	0.065
0.101	0.278
0.008	0.146
0.036	0.251
0.145	0.169
0.040	0.054
1.000	0.240
0.063	0.143
0.260	0.953
0.244	0.095
0.301	0.181
0.189	0.123
0.047	0.198
0.223	0.812
0.190	0.706
0.003	0.656
0.020	0.469

0.387	0.138
0.812	0.220
0.021	0.073
0.054	0.177
0.998	0.329
0.908	0.002
0.300	0.643
0.679	0.441
0.375	0.275
0.272	0.330
0.246	0.130
0.052	0.196
	0.197
	0.103
0.280	0.122
0.291	0.389
178	0.138
	0.896
	0.103
	0.142
	0.070
	0.442
	0.039
	0.227
	0.317
	0.458
	0.181
	0.974
	0.637
	0.044
	0.991
	0.915
	0.134
	0.262

0.011
0.101
0.113
0.077
0.118
0.769
0.441
0.907
0.306
0.072
0.019
0.541
0.106
0.065
0.133
0.102
0.124
0.447
0.292
0.477
0.139
0.134
0.003
0.101
0.077
0.038
0.082
0.339
0.200
0.442
0.207
0.021
0.009
0.258

0.030
0.022
0.085
0.049
0.069
0.230
0.197
0.272
0.042
0.114
0.450
0.136
0.386
0.514
0.677
0.707
0.980
0.085
0.009
0.055
0.206
0.001
0.810
0.867
0.318
0.108
0.017
0.836
0.320
0.068
0.207
1.000
0.176
0.761

0.419
0.715
0.315
0.571
0.579
0.033
0.974
0.043
0.899
0.075
0.858
0.134
0.194
0.515
0.312
0.643
0.067
0.000
0.214
0.542
0.295
0.147
0.098
0.637
0.131
0.497
0.542
0.123
0.257
0.341
0.107
0.177
0.107
0.384

0.021
0.203
0.301
0.670
0.317
0.206
0.324
0.117
0.132
0.164
0.145
0.039
0.304
0.004
0.105
0.831
0.489
0.425
0.052
0.583
0.806
0.792
0.572
0.004
0.891
0.823
1.000
1.000
1.000
0.995
1.000
1.000
0.485
0.007

0.018
0.416
0.027
0.920
0.950
0.875
0.030
0.373
0.300
0.055
0.188
0.116
0.195
0.131
0.020
0.771
0.144
0.565
0.554
0.004
0.005
0.019
0.478
0.018
0.173
0.061
0.093
0.252
0.068
0.017
0.008
0.761
0.012
0.289

0.040
0.272
0.234
0.158
0.164
0.020
0.691
0.079
0.147
0.524
0.243
0.245
0.154
0.160
0.339
0.098
0.184
0.279
0.316
0.218
0.281
0.694
0.148
0.987
0.581
0.157
0.001
0.254
0.518
0.043
0.100
0.058
0.429
0.004

0.038
0.686
0.398
0.340
0.026
0.960
0.498
0.066
0.496
0.613
0.027
0.222
0.489
0.468
0.754
0.105
0.702
0.405
0.018
0.048
0.522
0.018
0.038
0.940
0.990
0.578
0.408
0.215
0.450
0.158
0.495
0.617
0.294
0.205

0.755
0.456
0.374
0.591
0.012
0.572
0.493
0.435
0.044
0.295
0.045
0.918
0.828
0.905
0.033
0.441
0.265
0.010
0.246
0.549
0.409
0.114
0.769
0.043
0.777
0.679
0.634
0.413
0.691
0.679
0.086
0.867
0.047
0.954

0.055
0.146
0.192
0.645
0.247
0.001
0.593
0.643
0.606
0.616
0.806
0.086
0.766
0.042
0.934
0.900
0.117
0.171
0.018
0.281
0.190
0.896
0.338
0.567
0.422
0.011
0.168
0.974
0.062
0.261
0.967
0.067
0.078
0.604

0.733
0.281
0.164
0.150
0.112
0.244
0.134
0.074
0.307
0.535
0.537
0.175
0.391
0.219
0.223
0.976
0.785
0.239
0.971
0.067
0.240
0.551
0.020
0.397
0.151
0.215
0.218
0.990
0.060
0.062
0.288
0.306
1.000
0.743

0.953
0.694
0.300
0.267
0.559
0.068
0.007
0.162
0.762
0.805
0.398
0.397
0.348
0.272
0.216
0.066
0.579
0.328
0.314
0.905
0.383
0.239
0.981
0.121
0.372
0.608
0.627
0.952
0.457
0.700
0.076
1.000
0.014
0.567

0.149
0.150
0.077
0.210
0.275
0.132
0.002
0.167
0.768
0.160
0.356
0.876
0.126
0.665
0.044
0.440
0.387
0.859
0.000
0.630
0.007
0.871
0.990
0.002
0.412
0.250
1.000
0.064
0.095
1.000
0.094
0.086
0.894
0.001

0.725
0.109
0.396
0.131
0.265
0.146
0.513
0.229
0.350
0.059
0.206
0.194
0.422
0.076
0.265
0.499
0.375
0.851
0.172
0.780
0.238
0.342
0.351
0.206
0.181
0.440
0.165
0.859
0.651
0.566
0.155
0.883
1.000
0.208

0.261
0.238
0.147
0.253
0.732
0.764
0.849
0.898
0.667
0.130
0.168
0.131
0.282
0.068
0.621
0.463
0.176
0.087
0.507
0.094
0.356
0.424
0.580
0.614
0.851
0.790
0.104
0.069
0.177
0.241
0.112
0.125
0.081
0.888

0.098
0.354
0.798
0.175
0.475
0.013
0.116
0.001
0.600
0.296
0.822
0.963
0.238
0.001
0.416
0.007
0.040
0.016
0.246
0.420
0.198
0.054
0.056
0.017
1.000
0.883
0.596
0.372
0.200
0.329
0.276
0.172
0.361
0.343

			0.395		
			0.229		
			0.133		
			1.000		
			0.366		
			0.304		
			714		

D. Between social units

	Year				
	2010	2011	2012	2013	2014
Proportion pairwise overlap of 95% MCPs	0.008	0.506	0.339	0.022	0.225
	0.012	0.036	0.007	0.004	0.141
	0.008	0.0153	0.078		0.264
	0.002	0.0068	0.223		0.013
		0.4327	0.001	0.013	0.101
		0.0706	0.389	0.013	0.536
	0.008	0.038	0.302	2	0.136
	0.004	0.011	0.163		0.32
	4	0.333	0.063		0.016
		0.015	0.126		0.353
		0.013	0.031		0.0002
		0.003	0.653		0.012
		0.003	0.316		0.117
		0.043	0.218		0.002
		0.017	0.162		0.25
		0.047	0.331		0.573
		0.063	0.102		0.021
		0.018	0.031		0.261
		0.008	0.375		0.098
		0.034	0.073		0.323

	0.183	0.227
	0.133	0.007
0.086	0.913	0.288
0.150	0.088	0.029
20	0.166	0.067
	0.295	0.619
	0.002	0.402
	0.103	0.083
	0.064	0.269
	0.006	0.678
	0.014	0.147
	0.044	0.975
	0.003	0.254
	0.055	0.338
	0.004	0.576
	0.005	0.551
	0.081	0.365
	0.309	0.356
	0.038	0.048
	0.061	0.0002
	0.114	0.206
	0.003	0.108
	0.18	0.105
	0.007	0.06
	0.03	0.185
	0.451	0.029
	0.192	0.179
	0.009	0.0002
	0.004	0.334
	0.009	0.028
	0.121	0.111
	0.097	0.082
	0.007	0.084
	0.02	0.008

0.254	0.124
0.012	0.068
0.055	0.066
0.202	0.536
0.039	0.136
0.007	0.32
0.194	0.016
0.062	0.353
0.041	0.0002
0.086	0.012
0.164	0.117
0.169	0.002
0.069	0.25
0.223	0.573
0.09	0.021
0.165	0.261
0.108	0.098
0.101	0.323
0.004	0.227
0.046	0.007
0.311	0.288
0.094	0.029
0.017	0.067
0.014	0.619
0.096	0.402
0.002	0.083
0.044	0.269
0.1	0.678
0.017	0.147
0.999	0.975
0.155	0.254
0.173	0.338
0.125	0.576
0.255	0.551

0.55	0.365
0.314	0.356
0.572	0.048
0.197	0.0002
0.277	0.206
0.096	0.108
0.002	0.105
0.324	0.06
0.378	0.185
0.023	0.029
0.421	0.179
0.208	0.0002
0.005	0.334
0.615	0.028
0.24	0.111
0.073	0.082
0.013	0.084
0.236	0.008
0.09	0.124
0.053	0.068
0.247	0.066
0.066	0.05
0.001	
0.009	0.209
0.164	0.209
0.435	110
0.644	
0.437	
0.002	
0.041	
0.178	
0.5	
0.2	
0.063	

0.772
0.006
0.107
0.587
0.198
0.037
0.046
0.192
0.759
0.002
0.006
0.068
0.002
0.131
0.051
0.22
0.326
0.025
0.367
0.206
0.053
0.375
0.185
0.134
0.004
0.235
0.077
0.038
0.031
0.61
0.338
0.302
0.109
0.196

0.01
0.1
0.012
0.161
0.261
0.452
0.066
0.506
0.165
0.105
0.201
0.085
0.247
0.347
0.328
0.788
0.582
0.63
0.919
0.097
0.771
0.869
0.011
0.707
0.656
0.065
0.197
0.001
0.137
0.338
0.165
0.094
0.086
0.0009

0.121
0.029
0.213
0.029
0.097
0.057
0.114
0.0037
0.006
0.01
0.18798
0.21188
200
